# In Vitro Nephrotoxicity Induced by Herb-Herb Interaction between Radix Glycyrrhizae and Radix Euphorbiae Pekinensis

**DOI:** 10.1155/2020/6894751

**Published:** 2020-04-27

**Authors:** Meng Chen, Di Geng, Xin Yang, Xiaoxuan Liu, Siqi Liu, Pengmin Ding, Yuesheng Pang, Min Du, Xiuhua Hu, Rufeng Wang

**Affiliations:** ^1^School of Life Sciences, Beijing University of Chinese Medicine, Yangguang South Street, Fangshan District, Beijing 102488, China; ^2^Department of Animal Science and School of Molecular Biosciences, Washington State University, Pullman, WA 99164, USA

## Abstract

Radix Glycyrrhizae (RG)-Radix Euphorbiae Pekinensis (REP) is a representative incompatible herbal pair of Eighteen Incompatible Medicaments (EIM) and has been disputed in clinical application for a long time. The present study was performed with the Madin-Darby canine kidney (MDCK) cell line using cell cytotoxicity assay, apoptosis detection, cell cycle measurement, reactive oxygen species (ROS) determination, and high content analysis (HCA) in combination with high-performance liquid chromatography (HPLC) fingerprint comparison to clarify whether RG and REP can be concomitantly used from the perspective of cytotoxicity, investigate the major correlated compounds, and elucidate the underlying mechanisms. The results showed that the toxicity of REP could be significantly enhanced through its concomitant use with RG in the ratio of 1 : 1, and this increased toxicity could be weakened with the further increased proportion of RG. 3,3′-di-*O*-methylellagic acid-4′-*O*-*β*-D-xylopyranoside (DEAX) and 3,3′-di-*O*-methylellagic acid (DEA) were shown to be mainly responsible for the toxicity induced by concomitant use of REP and RG. Both RG-REP decoctions and the above two compounds boosted cell apoptosis, cellular morphological change, ROS accumulation, and mitochondrial membrane potential (MMP) disruption. In conclusion, the incompatible use of RG and REP is conditionally established because of the bidirectional regulatory effect of RG, and the major compounds responsible for RG-REP incompatibility are DEAX and DEA, which result in toxicity through activation of mitochondria-dependent apoptosis induced by increased ROS production. This study provided a basis for understanding the incompatible use of RG and REP and the EIM theory.

## 1. Introduction

Drug-drug interaction is a very important aspect in the clinical practice and is a focus of medical research. In Chinese medicine, an ancient mysterious theory about drug contradiction, that is, the Eighteen Incompatible Medicaments (EIM), has always been a disputable issue of medical practitioners. This theory discourages the concomitant use of eighteen herbal drug pairs, such as Radix Glycyrrhizae (RG) and Radix Euphorbiae Pekinensis (REP), Radix Aconiti and Bulbus Fritillariae Cirrhosae, and Radix et Rhizoma Veratri and Radix Ginseng. Incompatible medicaments in Chinese medicine stemmed from the famous Shennong's *Herbal Classic*—the extant earliest classic book of Traditional Chinese Medicine (TCM), which abstractly proposed this concept. Subsequently, a book entitled *Variorum of Shennong's Herbal Classic* authored by Hongjing Tao, who was a Chinese pharmacologist in the Northern and Southern Dynasties, listed the incompatible herbal drug pairs including those mentioned above. Although these incompatible herbal drug pairs have been avoided as much as possible by TCM practitioners since ancient times, the experience of concomitant use of these pairs has been accumulated, and the treatment cases of the rare and intractable diseases using them have also been recorded now and then [1–3]. Pharmacologists possess different viewpoints on this concept and have not yet come to an agreement [4, 5]. Therefore, this theory has been observed and disputed by Chinese pharmacologists since ancient times, and it is still an interesting question whether EIM should be absolutely avoided in the clinical practice and whether there has been scientific basis to support this occult theory.

REP and RG constitute one of such herbal pairs of EIM. REP is the dried roots of *Euphorbia pekinensis* Rupr., which is a well-known poisonous plant of genus *Euphorbia* in the Euphorbiaceae family. This herbal drug has been officially recorded in Chinese pharmacopoeia [6] for the treatment of oedema, malignant ascites, and urinary retention. In the clinic, REP exhibits explicit therapeutic effects on relieving constipation, purging fluid, and eliminating phlegm [7, 8]. Modern pharmacological investigations also demonstrated that its extract exhibited a variety of biological effects, including antineoplastic, antiviral, and analgesic activities [9–11]. RG, which comes from the dried roots of either *Glycyrrhiza uralensis* Fisch. or *G. glabra* L. or *G. inflate* Bat., is one of the most widely used nourishing herbal medicines in TCM. It is usually used in combination with other herbs and prescribed in approximately 60% of TCM formulas because most TCM practitioners believe that RG has the capacity to enhance the efficacy of other ingredients and reduce toxicity [12]. Meanwhile, RG has been used as a food supplement in other countries, such as Japan and the UK [13–16]. It is generally believed that concomitant use of these two herbal drugs should be forbidden in the clinic. There has been experimental evidence showing that the concomitant use of REP and RG results in more severe adverse effects on the tissues of the kidney, heart, and liver of experimental animals than separate use and leads to significantly increased levels of alanine aminotransferase, phosphocreatine kinase, lactate dehydrogenase, *γ*-hydroxybutyrate dehydrogenase, urea, and creatinine [17]. Furthermore, a study reported that combination of RG and REP caused vascular congestion and lymphocytic infiltration in the renal and hepatic tissues of rats, indicating some toxic and side effects [18]. This reminds us that the concomitant use of both drugs may be harmful to animals because of the generation or increment of toxic compounds. However, to our knowledge, there is limited evidence proving which compound/compounds is/are related to the increased nephrotoxicity of this herbal pair. In order to obtain the direct evidence of the underlying toxic mechanism induced by concomitant use of RG and REP, we carried out the study to investigate the correlation between the toxicity and compounds in RG-REP decoctions on the basis of chemical composition change resulting from a combination process.

## 2. Materials and Methods

### 2.1. Materials, Reagents, and Instruments

RG and REP were purchased from Anguo Chinese crude drug market in Hebei Province of China, and botanical identification was done by Prof. Rufeng Wang. Two voucher specimens (Nos. 201205041 and 201205042 for RG and REP, respectively) have been deposited at the herbarium of School of Life Sciences, Beijing University of Chinese Medicine, China. Reference compounds 3,3′-di-*O*-methylellagic acid-4′-*O*-*β*-D-xylopyranoside (DEAX) and 3,3′-di-*O*-methylellagic acid (DEA) were prepared in our laboratory, and their purities were determined to be >98% by high-performance liquid chromatography (HPLC).

Dulbecco's modified Eagle's medium (DMEM), fetal bovine serum (FBS), and trypsin were supplied by Gibco Invitrogen Corp. (Grand Island, CA, USA). 3-(4,5-dimethyl-2-thiazolzyl)-2,5-diphenyl-2-*H*-tetrazolium bromide (MTT) and dimethyl sulfoxide (DMSO) were products purchased from Sigma Chemical Co. (Deisenhofen, Germany). Cell culture flasks, 96-well plates, 6-well plates, penicillin, and streptomycin were purchased from Corning Inc. (Cambridge, MA, USA). Rhodamine 123, Hoechst 33342, 2′,7′-dichlorodihydrofluorescein diacetate (DCFH-DA), propidium iodide (PI), ethylenediaminetetraacetic acid (EDTA), and Annexin V-fluorescein isothiocyanate/PI (Annexin V-FITC/PI) apoptosis detection kit were obtained from Beijing Aoboxing Biotechnology Co., Ltd. (Beijing, China). A caspase-3/7 assay kit and cell cycle kit were supplied by EMD Millipore Co., Ltd. (Temecula, CA, USA). Acetonitrile of HPLC grade used for HPLC analysis was produced by Fisher Co. (Pittsburgh, PA, USA). Deionized water was prepared using a Millipore water purification system made by Millipore Corporation (Billerica, MA, USA).

A Bio-Rad Model 550 microplate reader was produced by Bio-Rad Laboratories (Hercules, CA, USA). A Nikon TE2000 microscope equipped with a high-resolution digital camera (Nikon DXM 1200F) was manufactured by Nikon Instruments, Inc. (Tokyo, Japan). A CytoFLEX Flow Cytometer was the product of Beckman Coulter (Brea, CA, USA). A Muse Cell Analyzer was obtained from Merck Millipore Corp. (Billerica, MA, USA). An Opera Phenix High Content Analysis (HCA) confocal microscope equipped with a 20× Plan Apo objective lens was from PerkinElmer Inc. (Boston, MA, USA). A Waters 1500 series liquid chromatograph equipped with a 1525 binary pump, an on-line degasser, a manual injector, and a 2489 UV/visible detector were the products of Waters Corp. (Milford, MA, USA).

### 2.2. Cell Line and Treatment

The Madin-Darby canine kidney (MDCK) cell line was supplied by the Cell Resource Center, Peking Union Medical College (CRC/PUMC, China) and cultured in DMEM containing 10% FBS and 1% penicillin-streptomycin in an atmosphere of 5% CO_2_ and 95% air at 37°C and constant humidity. The cells were inoculated in the 96-well or 6-well plates and cultured overnight prior to treatment with each RG-REP decoction or each compound.

### 2.3. Sample Preparation

The crude drug material of RG and REP was separately pulverized and passed through a 40-mesh sieve and then used for decoction preparation. Individual decoctions and combined decoctions which could be further divided into combination ratios including 1 : 1, 2 : 1, and 3 : 1 (RG : REP, counted by weight of crude drugs) were prepared according to Table 1. In brief, the powdered material for each decoction was extracted twice with 10 volumes (*v*/*w*) of distilled water by boiling for 0.5 h each, and the lost water was made up at any time; then, the extract was combined, filtered, and concentrated to a solution containing 0.1 g of REP per mL. The resultant solution was diluted to the desired concentrations (0.4, 0.7, 1.0, 1.3, 1.6, and 1.9 mg of REP per mL) using DMEM prior to treating MDCK cells.

For DEA and DEAX, they were accurately weighted and dissolved in DMSO, respectively. Then, they were diluted with DMEM to allow the final concentration of DMSO in the culture medium less than 1.0% (*v*/*v*). As such, the maximum concentrations of DEA and DEAX dissolved in DMEM were 300 and 500 *μ*M, respectively.

### 2.4. Cell Cytotoxicity Assay

The inhibition rates of decoctions or compounds on MDCK cells were detected using MTT assay. The cells were seeded into 96-well plates at a density of 3 × 10^4^ cells per well and cultured at 37°C for 24 h before being exposed to decoctions or compounds at various concentrations. After being treated for 48 or 24 h, the supernatant was removed, and the cells were incubated with 100 *μ*L of MTT solution for 4 h under cell culture conditions. Then, the liquid part was discarded, and the cells were dissolved in 150 *μ*L of DMSO and shaken for 10 min to dissolve the formazan crystal generated. After that, the absorbance (*A*) value of the resultant solution was measured on a microplate reader at the wavelength of 570 nm. The cell inhibition rates were calculated by the following formula: cell inhibition rate (%) = (1 − experimental group′s *A*/control group′s *A*) × 100%. The concentrations of 50% toxicity (TC_50_) on MDCK cells were also determined. The MDCK cellular morphology was observed with a Nikon TE2000 microscope, and three different fields per well were randomly chosen and photographed.

### 2.5. Cell Apoptosis Detection

The percentages of survival, early apoptotic, late apoptotic, and necrotic cells were detected using a caspase-3/7 kit or Annexin V-FITC/PI apoptosis detection kit. First, MDCK cells at the density of 1.8 × 10^5^ cells per well were cultured in 6-well plates for 24 h and then treated with each RG-REP decoction for 48 h or each compound for 24 h. After treatment, the cells were trypsinized with 0.05% trypsin without EDTA and then centrifuged in PBS three times. Subsequently, 5 *μ*L of Muse caspase-3/7 working solution or 5 *μ*L of Annexin V-FITC and 150 *μ*L of 7-AAD working solution or 5 *μ*L of PI solution were successively added in the dark at room temperature to stain the cells for 15 min. After that, the stained cells were detected with a Muse Cell Analyzer or a CytoFLEX Flow Cytometer within 1 h. For each analysis, a total of 10,000 events were recorded.

### 2.6. Cell Cycle Measurement

The cell cycle distribution of MDCK cells was analyzed using a cell cycle kit. Briefly, MDCK cells at the density of 1.8 × 10^5^ cells per well were seeded in 6-well plates for 24 h and then treated with each RG-REP decoction. About 48 h later, the cells were dissociated and harvested through centrifugation at 1500 rpm for 5 min. Then, they were permeabilized in 70% ethanol at 4°C for 24 h and washed three times with PBS. The pellets were lysed in 100 *μ*L of cell cycle reagent in the dark for 30 min and examined using the Muse Cell Analyzer. At least 10,000 events of MDCK cells were typically acquired for each analysis.

### 2.7. Intracellular Reactive Oxygen Species (ROS) Production Measurement

The intracellular ROS production was measured using DCFH-DA dye as described by the literature [19]. The MDCK cells at the density of 1.8 × 10^5^ cells per well were first seeded into 6-well plates and incubated overnight. Next, they were exposed to various concentrations of each RG-REP decoction for 48 h or each compound for 24 h. Then, the cells were harvested using 0.05% trypsin without EDTA and centrifuged at 1500 rpm for 5 min. After that, they were resuspended in a buffer containing 10 *μ*M DCFH-DA at 37°C in the dark for 30 min and washed twice with PBS to remove unincorporated dye. Finally, the fluorescence intensity of the samples was immediately recorded on the CytoFLEX Flow Cytometer at the excitation and emission wavelengths of 485 and 530 nm, respectively. A minimum of 10,000 events were collected on each sample.

### 2.8. High Content Analysis

The cell appearance and average fluorescence intensity were monitored by HCA coupled with Hoechst 33342/PI/Rhodamine 123 staining assay. The cells were first seeded into 96-well plates at the density of 3 × 10^4^ cells per well. After incubation for 24 h, the medium was removed, and cells were treated with the medium containing each RG-REP decoction or each compound. About 48 or 24 h later, they were stained in the dark with PBS containing 5 *μ*M Hoechst 33342, 10 *μ*M PI, and 10 *μ*M Rhodamine 123, under cell culture conditions for 45 min. The stained cells were washed twice with PBS and maintained in prewarmed DMEM without phenol red for live cell imaging. Finally, the MDCK cells were imaged in 96-well plates using an Opera Phenix HCA confocal microscope equipped with a 20× Plan Apo objective lens (NA 0.45) at three fixed excitation and emission wavelengths for Hoechst 33342 (Ex/Em 350/461 nm), PI (Ex/Em 535/615 nm), and Rhodamine 123 (Ex/Em 507/529 nm). Nine fields per well were taken to cover the entire well.

### 2.9. HPLC Analysis

The HPLC fingerprints of each decoction and content assay of DEAX and DEA were obtained on a Waters 1500 series chromatograph. Each decoction sample was appropriately diluted to the final concentration of 0.5 mg of REP per mL and filtered through a 0.22 *μ*m membrane filter. Then, each 10.0 *μ*L of the samples was loaded into a Thermo Hypersil BDS C_18_ column (250 mm × 4.6 mm, 5 *μ*m) at the column temperature of 30°C. The chromatography was run at a flow rate of 1.0 mL/min using acetonitrile as mobile phase A and 1.0% acetic acid in purified water as mobile phase B with gradient elution program including 2–8% A (0–6 min), 8–13% A (6–21 min), 13–20% A (21–30 min), 20–35% A (30–40 min), 35–45% A (40–50 min), 45–75% A (50–60 min), 75–90% A (60–65 min), and 90–100% A (65–70 min). The stock solutions of DEAX (0.02 *μ*g/mL) and DEA (40 *μ*g/mL) were prepared with methanol and further diluted into a series of working solutions for the establishment of calibration curves. The calibration curves for DEAX and DEA were obtained by plotting their peak area (*Y*) versus amount (*X*, in *μ*g). As a result, the regression equations and coefficient correlations (*r*) for DEAX and DEA were *Y* = 2.80374 × 10^5^*X* + 1644.7 (*r* = 0.9996, 0.00156–0.4 *μ*g) and *Y* = 4 × 10^8^*X* + 28851 (*r* = 0.9997, 0.00200–20 *μ*g), respectively.

### 2.10. Data Analysis

The results presented in this study were the averages of at least three replicates and were presented as means ± SD. Statistical analysis was carried out by SPSS 19.0, and statistical significance was evaluated by Student's *t*-test for comparison of the mean values. *P* < 0.05 was selected as the criteria for statistical significance.

## 3. Results

### 3.1. The Cytotoxicity of REP Was Increased when Coused with RG at the Ratio of 1 : 1, and This Effect Was Alleviated along with the Increment of RG Dose

**Figure 1:**
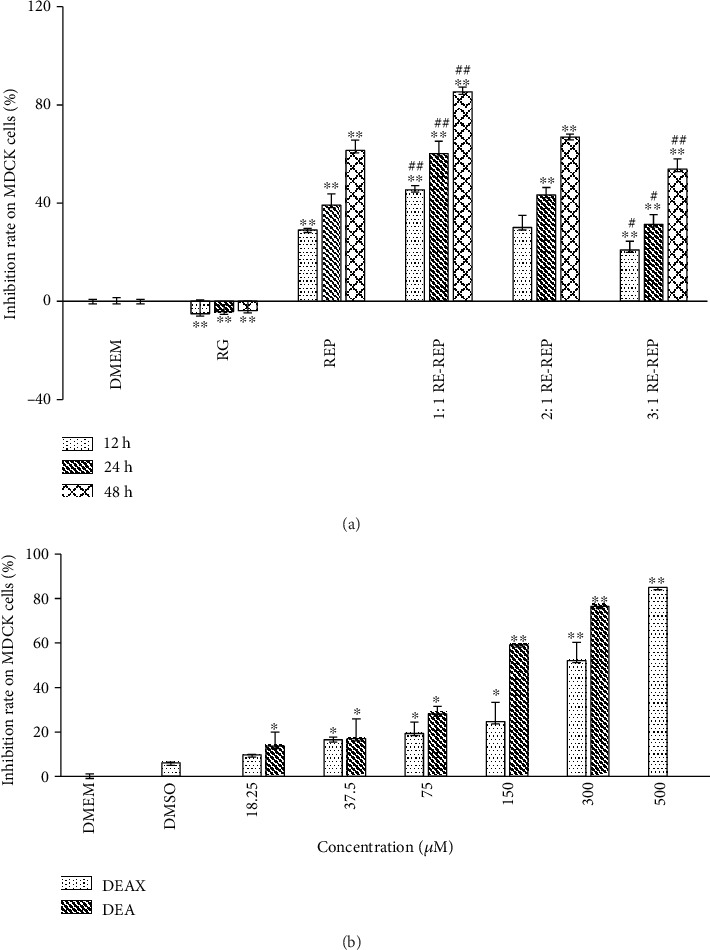
The influence of the RG-REP decoctions and the compounds on inhibition rate of MDCK cells. (a) Shows the inhibition rates of decoctions at the representative concentration of 1.0 mg/mL; (b) shows the inhibition rates of MDCK cells treated with two compounds at a series of concentrations for 24 h. All data are presented as means ± SD, *n* = 3. ^∗^0.01 < *P* < 0.05 and ^∗∗^*P* < 0.01, compared with the DMEM control group; ^#^0.01 < *P* < 0.05 and ^##^*P* < 0.01, compared with the REP group.

The results obtained from the MTT assay showed that the inhibition rate of the RG decoction on MDCK cells was decreased even though without significant difference compared with that of the DMEM control group, suggesting RG was nontoxic to MDCK cells. In contrast, the cytotoxicity of REP and three combined decoctions to MDCK cells was more prominent compared with that of DMEM control group. What is more, a significant dose- and time-dependent relationship was established. With the same concentration, the 1 : 1 RG-REP decoction was more cytotoxic than the REP decoction at all time points tested. Interestingly, the 3 : 1 RG-REP decoction provided significant protective effect on the cells compared with the REP decoction and the 1 : 1 RG-REP decoction. The inhibition rate of the 2 : 1 RG-REP decoction on the cells was between those of the 1 : 1 and 3 : 1 RG-REP decoctions (Figure 1(a) and Supplementary Figure 1). Quantitatively, the TC_50_ values of REP, 1 : 1, 2 : 1, and 3 : 1 RG-REP decoctions on MDCK cells at 12, 24, and 48 h were ranged from 1.147 to 4.074, from 0.796 to 2.649, and from 0.452 to 1.736 mg/mL, respectively.

### 3.2. 1 : 1 RG-REP Decoction Boosted Cellular Morphological Change, Apoptosis, Mitochondrial Membrane Potential (MMP) Disruption, and ROS Accumulation

**Figure 2:**
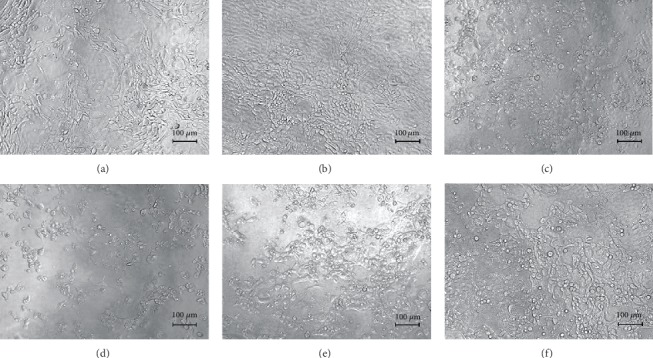
The influence of the RG-REP decoctions on morphology of MDCK cells (100×). (a–f) Show the morphology of MDCK cells under a light microscope after MDCK cells were treated with DMEM, RG, REP, 1 : 1 RG-REP, 2 : 1 RG-REP, and 3 : 1 RG-REP at 1.0 mg/mL for 48 h.

The effect of each RG-REP decoction on MDCK cells was confirmed by observing cellular morphology under a light inverted microscope.  Figure 2 shows the representative images of MDCK cells in an untreated control group and the groups treated with each RG-REP decoction at the concentration of 1.0 mg/mL for 48 h. In comparison with that in the DMEM control group, the cell density in the RG decoction group increased, while that in the REP decoction group obviously decreased. The cell morphology of MDCK cells in the REP group became smaller and round shaped. The lowest cell density was observed in the 1 : 1 RG-REP decoction group. Nevertheless, the increasing cell density was observed along with the proportion increment of RG, i.e., in the 2 : 1 and 3 : 1 RG-REP decoction groups (Figure 2).

**Figure 3:**
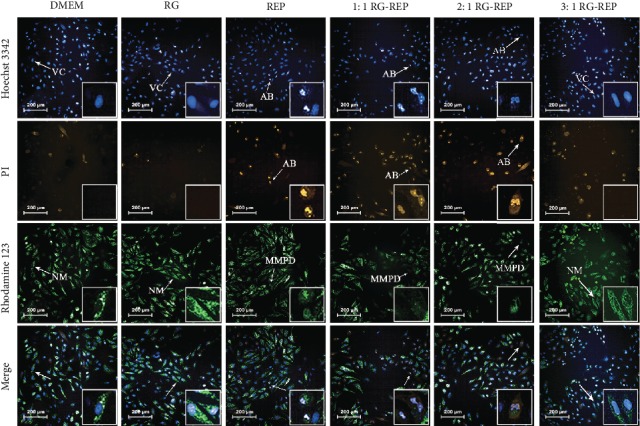
The effects of RG-REP decoctions on cell death of MDCK cells (200×). Representative images were obtained using an HCA coupled with Hoechst 33342/PI/Rhodamine 123 staining assay. VC: viable cell; AB: apoptotic body; NM: normal mitochondrion; MMPD: MMP disruption.

The results of HCA coupled with Hoechst 33342/PI/Rhodamine 123 staining assay further confirmed the cytotoxic effect of each RG-REP decoction on morphological features of MDCK cells. As shown in Figure 3, the majority of cells in the RG group displayed homogeneous blue nuclei (Hoechst 33342 positive) and bright green cytoplasm (Rhodamine 123 positive), and few of them exhibited red nuclei (PI positive). This demonstrated that most of the cells were viable (VC) and not in the state of apoptosis or necrosis. In contrast, in the REP group and the 1 : 1 RG-REP group, an increased number of deep red nuclei and apoptotic bodies (AB) were observed, which are the features of late apoptosis or necrosis.

The morphological change of mitochondria firmly convinced us of the toxicity of RG-REP decoctions. The normal mitochondria (NM) which exhibited the morphological features that were well-structured, interconnected, and rod-shaped were enriched in the DMEM control group and the RG group, whereas the mitochondria in the groups of the REP decoction and the 1 : 1 RG-REP decoction were dot-like round for MMP disruption. These results implied that the REP decoction and the 1 : 1 RG-REP decoction might promote cell apoptosis or necrosis.

**Figure 4:**
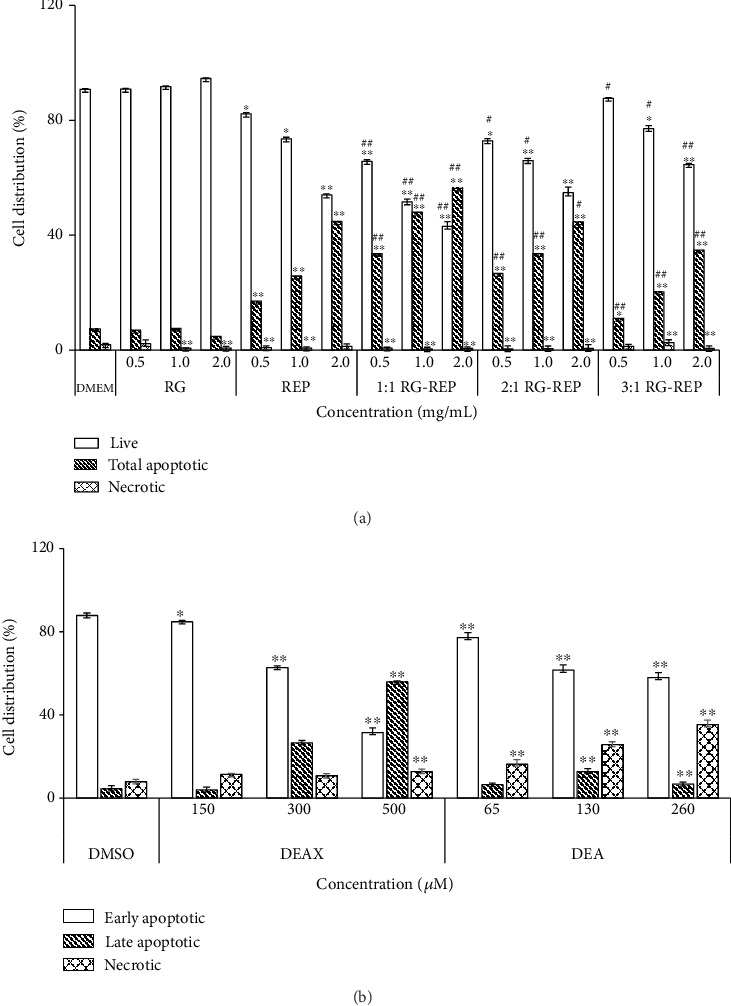
Quantitative analysis of the percentages of living, apoptotic, and necrotic cells. (a) Shows the distribution of MDCK cells incubated with various concentrations of each RG-REP decoction for 48 h; (b) shows the distribution of MDCK cells incubated with various concentrations of DEAX and DEA for 24 h. Data are presented as means ± SD, *n* = 3. ^∗^0.01 < *P* < 0.05 and ^∗∗^*P* < 0.01, compared with the DMEM (or vehicle) control group; ^#^0.01 < *P* < 0.05 and ^##^*P* < 0.01, compared with the REP group.

**Figure 5:**
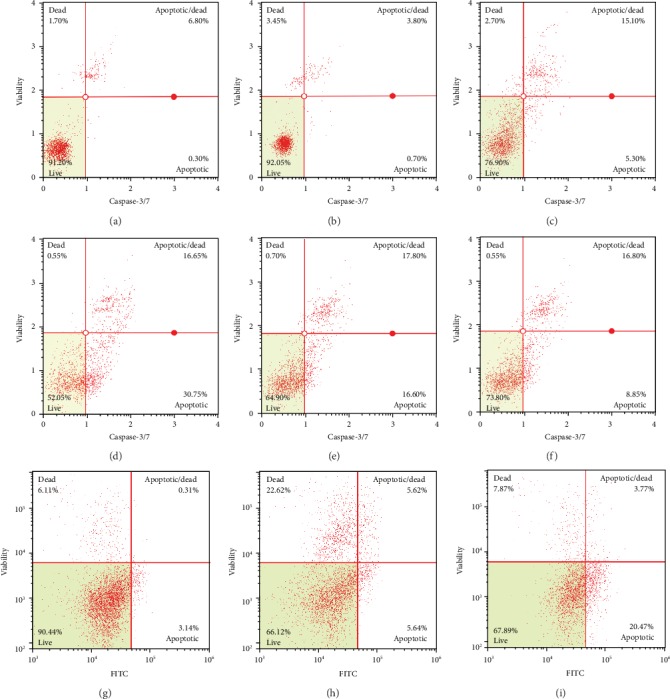
Representative flow-cytometry scatter plot of MDCK cells. (a–f) Show the percentages of alive (left lower quadrant), early apoptotic (right lower quadrant), late apoptotic (right upper quadrant), and dead (left upper quadrant) cells after they were treated with DMEM, RG, REP, 1 : 1 RG-REP, 2 : 1 RG-REP, and 3 : 1 RG-REP at 1.0 mg/mL for 48 h. (g–i) show the percentages of the 4 types of cells mentioned above after being treated with 1.0% DMSO, 130 *μ*M DEA, and 300 *μ*M DEAX for 24 h.

The percentages of alive, early apoptotic, late apoptotic, and dead cells quantitatively measured by flow cytometry revealed the cell death manner primarily contributed to the cytotoxicity of RG-REP decoctions. As shown in Figures 4(a) and 5, treatment with RG did not stimulate significant cell apoptosis. In contrast, treatment with REP or three combined RG-REP decoctions significantly increased the percentage of total apoptotic cells in a dose-dependent manner. Furthermore, the percentage of total apoptotic cells in the REP decoction group at the concentration of 1.0 mg/mL was 25.8%, whereas it increased to 47.9% in the 1 : 1 RG-REP decoction group and decreased again to 20.43% when the ratio of RG-REP increased to 3 : 1. As for the percentages of necrotic cells in three combined RG-REP decoction groups which ranged from 0.47 to 2.57%, almost no significant difference was observed compared to the REP decoction group which ranged from 0.73 to 1.30%. In general, the percentage of total apoptotic cells was 7.92–102.7 times as many as the percentage of necrotic cells. These results confirmed that REP decoction and 1 : 1 RG-REP decoction preferably promoted cell apoptosis, and the latter was stronger than the former.

**Figure 6:**
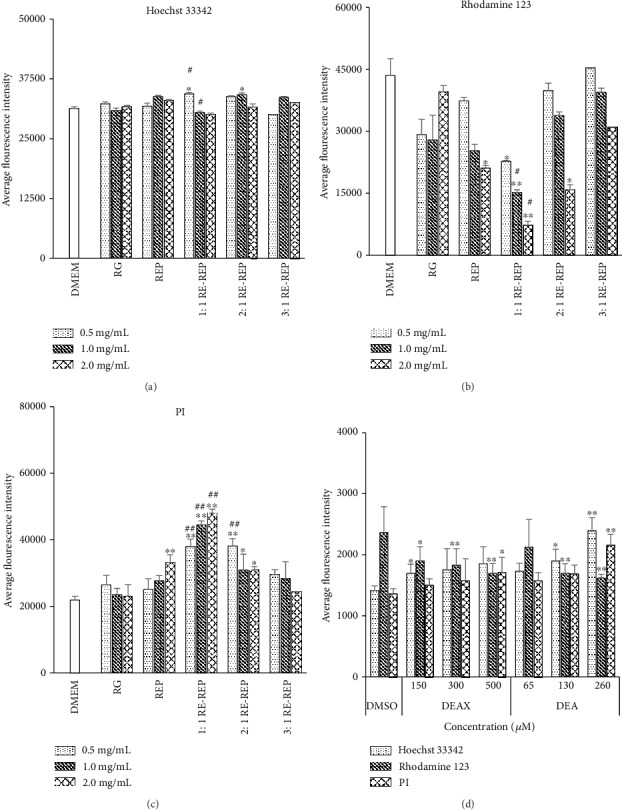
The influence of RG-REP decoctions and compounds on fluorescence intensity of Hoechst 33342/Rhodamine 123/PI. (a) Shows the influence of each RG-REP decoction on the fluorescence intensity of Hoechst 33342; (b) shows the influence of each RG-REP decoction on the fluorescence intensity of Rhodamine 123; (c) shows the influence of each RG-REP decoction on the fluorescence intensity of PI; (d) shows the influence of DEAX and DEA on the fluorescence intensity of Hoechst 33342, Rhodamine 123, and PI. The results are presented as means ± SD, *n* = 3. ^∗^0.01 < *P* < 0.05 and ^∗∗^*P* < 0.01, compared with the DMEM (or vehicle) control group; ^#^0.01 < *P* < 0.05 and ^##^*P* < 0.01, compared with the REP group.

The results of HCA through detection of the fluorescence intensity of Rhodamine 123 also made clear that MMP disruption was involved in the cell damage induced by the REP and combined RG-REP decoctions. The fluorescence intensity of Rhodamine 123, which is a good indicator for MMP, was not changed significantly by RG at all experimental concentrations and REP at the low concentrations of 0.5 and 1.0 mg/mL. However, the groups treated with REP at the high concentration of 2.0 mg/mL and 1 : 1 RG-REP decoction at all experimental concentrations displayed significant lower fluorescence intensity compared with that of the DMEM control group. In addition, the fluorescence intensity significantly decreased from 37942.7 to 25002.2, from 44486.35 to 27652.2, and from 48004.7 to 33225.5 compared with that of the REP decoction group after treatment with 1 : 1 RG-REP decoction at the concentrations of 0.5, 1.0, and 2.0 mg/mL, respectively (Figure 6(c)). These results indicated that the apoptosis of MDCK cells was ascribed to the mitochondrial dysfunction.

**Figure 7:**
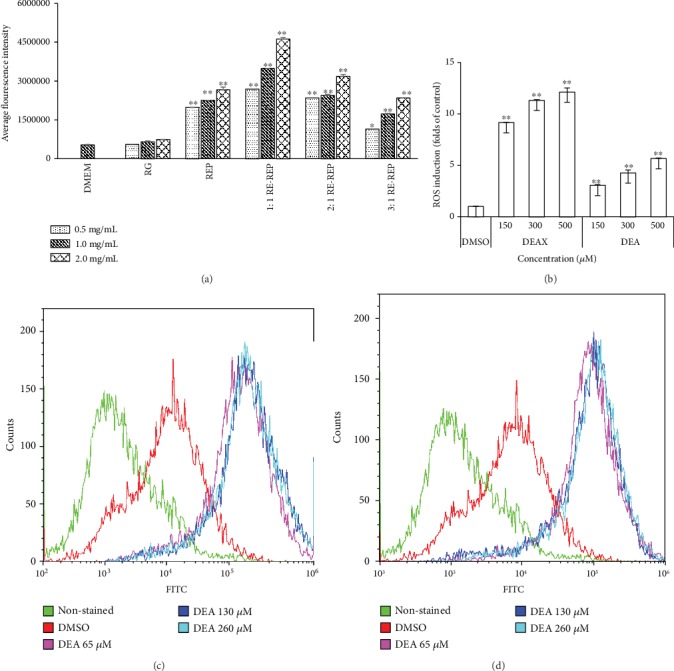
The effects of RG-REP decoctions and compounds on ROS generation. (a) Shows the influence of decoctions on fluorescence intensity of DCF; (b) shows the influence of DEAX and DEA on ROS accumulation; (c, d) show the histograms of the fluorescence intensity of DCF after treatment with DEAX and DEA for 24 h. The results are presented as means ± SD, *n* = 3. ^∗^0.01 < *P* < 0.05 and ^∗∗^*P* < 0.01, compared with the DMEM (or vehicle) control group.

Since mitochondria are both the primary source and the target of ROS [20, 21], so the ROS changes were determined. As shown in Figure 7(a), RG did not affect the fluorescence intensity of 2′,7′-dichlorofluorescein (DCF), which is usually used to reflect the amount of ROS, even at the high concentration of 2.0 mg/mL. However, the generation of ROS increased in a concentration-dependent manner after the MDCK cells were treated with REP and three combined decoctions for 48 h. Moreover, ROS induced by the 1 : 1 RG-REP decoction at the concentrations of 0.5, 1.0, and 2.0 mg/mL increased by approximately 5.05–8.68-folds compared with that of the DMEM control group. It was also increased 135.5, 153.8, and 173.9% compared with that of the REP decoction group at the same concentrations. In contrast, the level of intracellular ROS was significantly suppressed in the 3 : 1 RG-REP decoction group compared with that of the REP decoction group. The DCF fluorescence intensity of the 2 : 1 RG-REP decoction group was between those of the 1 : 1 and 3 : 1 RG-REP decoction groups. The above results demonstrated that the accumulation of ROS played an important role in the apoptosis of MDCK cells induced by the 1 : 1 RG-REP decoction.

**Figure 8:**
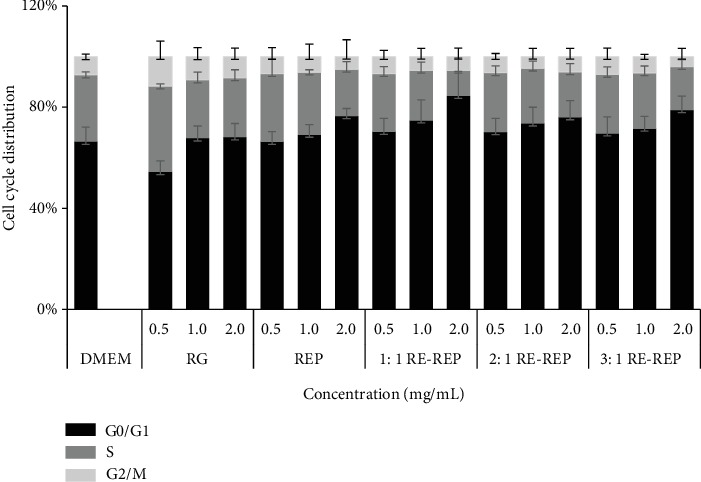
The effects of RG-REP decoctions on cell cycle distribution.

ROS has been reported to play an essential role in cell cycle progression [22], which led us to investigate whether each RG-REP decoction treatment affects the cell cycle using a cell cycle kit. The results showed that the percentage of cells at the G0/G1 phase in all groups increased in a dose-dependent manner after being exposed to RG-REP decoctions for 48 h. In the 1 : 1 RG-REP decoction group, it was 67.68, 72.64, and 82.52% at the concentrations of 0.5, 1.0, and 2.0 mg/mL, respectively, although no significant difference was observed compared to the REP decoction group. The cell population at the S phase was subsequently continued with a decrease although the difference was not significant (Figure 8).

### 3.3. The Contents of DEAX and DEA in 1 : 1 RG-REP Decoction Were Higher than Those in REP Decoction and Decreased along with the Increment of RG

**Figure 9:**
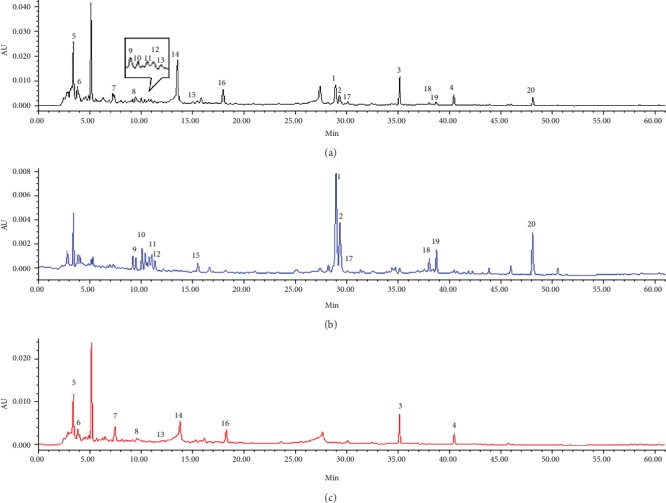
Comparison of HPLC fingerprints obtained with decoctions. (a) Shows the fingerprint of 1 : 1 RG-REP decoction with 20 characteristic peaks; (b) shows the fingerprint of RG decoction with 11 peaks out of the above 20 peaks; (c) shows the fingerprint of REP decoction with 9 peaks out of the above 20 peaks. Peaks 3 and 4 were identified as DEAX and DEA, respectively.

**Figure 10:**
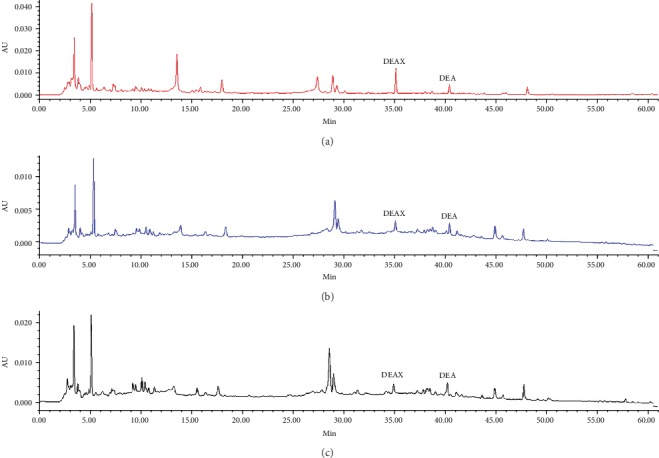
Quantitative comparison of DEAX and DEA in RG-REP decoctions by HPLC. (a–c) Show the peaks of DEAX and DEA in HPLC fingerprints obtained with an equal quantity of 1 : 1, 2 : 1, and 3 : 1 RG-REP decoctions, respectively.

**Figure 11:**
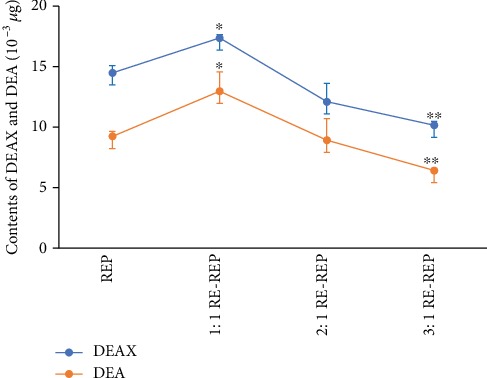
The contents of DEAX and DEA in RG-REP decoctions. The results are presented as means ± SD, *n* = 3. ^∗^0.01 < *P* < 0.05 and ^∗∗^*P* < 0.01, compared with the REP group.

The HPLC fingerprints obtained with individual decoctions and combined RG-REP decoctions were compared to identify the compounds responsible for the cytotoxicity. Two compounds corresponding to peaks 3 and 4 in Figures 9 and 10, which were from REP and identified as DEAX and DEA, were found to be quantitatively different between the REP decoction and the three combined decoctions. The contents of these two compounds in the 1 : 1 RG-REP decoction were higher than those in the REP decoction, and they decreased along with the increment of RG. That is to say, the contents of DEAX and DEA were the highest in the 1 : 1 RG-REP decoction and then successively decreased in the 2 : 1 and 3 : 1 RG-REP decoctions (Figure 11). The content variation tendency of these two compounds in the experimental groups was consistent with that of the cytotoxicity. This implied that the increased cytotoxicity of the 1 : 1 RG-REP decoction might be attributed to these two compounds.

### 3.4. Effect of DEAX and DEA on Cellular Morphology, Apoptosis, MMP Disruption, and ROS Generation

The pivotal role of DEAX and DEA in the increased cytotoxicity of the 1 : 1 RG-REP decoction was confirmed by the tests of cell inhibition rate, cellular morphological change, apoptosis, MMP, and ROS generation.

As shown in Figure 1(b), DEAX and DEA significantly inhibited MDCK cells in a concentration-dependent manner. Compared with the vehicle control (DMSO) group, the inhibitory effects were significantly increased after treatment with 37.5 *μ*M DEAX or 18.25 *μ*M DEA. Besides, MDCK cells were more sensitive to DEA than to DEAX at the same concentration. DEAX and DEA hereby induced cytotoxicity to MDCK cells with the TC_50_ values of approximately 297.4 and 128.3 *μ*M, respectively.

**Figure 12:**
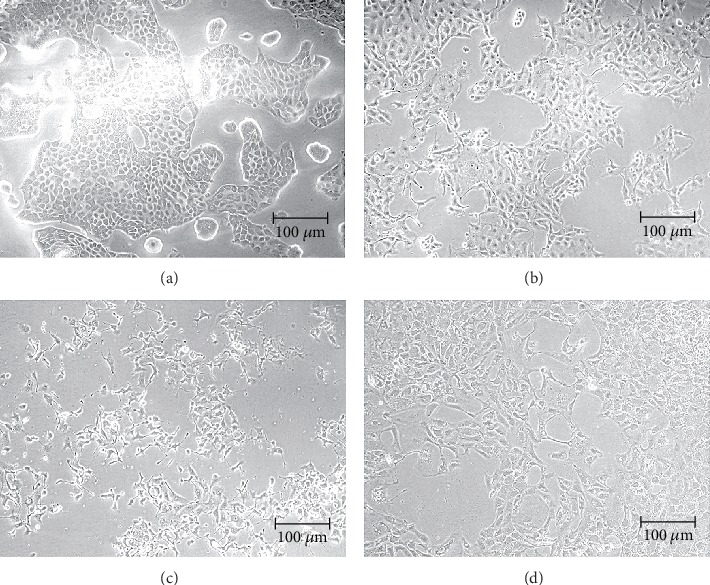
The influence of DEAX and DEA on morphology of MDCK cells (100×). (a–d) Show the morphology of MDCK cells under a light microscope after they were treated with 1.0% DMSO, 60 *μ*M cisplatin, 130 *μ*M DEA, and 300 *μ*M DEAX for 24 h, respectively.

**Figure 13:**
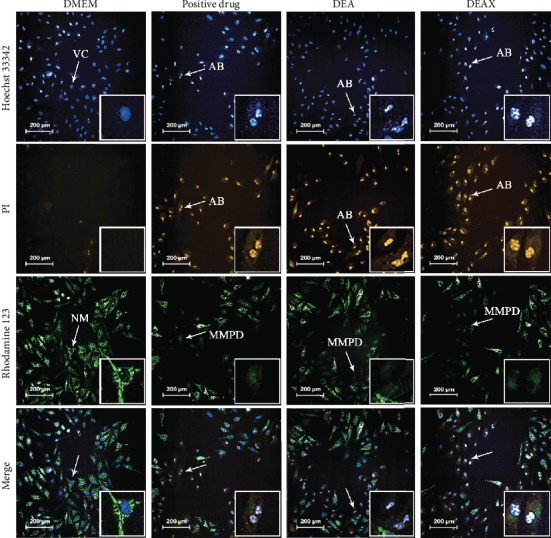
The effects of the compounds on cell death of MDCK cells (200×). Representative images were obtained using an HCA coupled with Hoechst 33342/PI/Rhodamine 123 staining assay. VC: viable cell; AB: apoptotic body; NM: normal mitochondrion; MMPD: MMP disruption.

The micromorphology of MDCK cells after treatment with 250 *μ*M DEAX and 150 *μ*M DEA for 24 h (Figure 12) was consistent with that of the cytotoxicity (Figure 1(b)). The cells in the DMSO control group grew into a confluence monolayer and exhibited a normal spindle shape. However, the cells in the DEAX and DEA groups displayed obvious damages, such as cytoplasm shrinkage, membrane blebbing, and cell-cell separation increment. The damages in these two groups were similar to those induced by cisplatin, a nephrotoxic positive drug, at the concentration of 60 *μ*M. The cellular morphology observed by HCA also showed that an increased number of cells with apoptotic bodies (AB) and disrupted MMP were observed after treatment with DEAX or DEA, while the number of viable cells (VC) was significantly less than that in the DMSO control group (Figure 13).

The exact percentage of apoptotic cells was determined by Annexin V-FITC/PI labelling assay. As shown in Figures 4(b) and 5, the percentage of the apoptotic cells in the DMSO control group was 3.43 ± 0.07%, whereas it changed to 2.88 ± 0.33, 24.89 ± 0.76, and 60.22 ± 0.64%, respectively, after treatment with 150, 300, and 500 *μ*M DEAX for 24 h. These data corresponded with 0.35-, 2.99-, and 5.16-folds of those of necrotic cells. As for DEA, exposing MDCK cells to 130 and 260 *μ*M for 24 h obviously increased the percentages of apoptotic and necrotic cells compared with the DMSO control group. What is more, the percentages of necrotic cells after treatment with 65, 130, and 260 *μ*M DEA for 24 h corresponded with 4.32-, 2.10-, and 3.77-folds of those of apoptotic cells, respectively.

As shown in Figure 6(d), the Rhodamine 123 fluorescence intensity of cells treated with 150, 300, and 500 *μ*M DEAX corresponded with 80.29, 77.39, and 71.71% of those of cells in the DMSO control group. Similarly, the fluorescence intensity of cells treated with 65, 130, and 260 *μ*M DEA corresponded with 89.8, 71.6, and 68.4% of that of cells in the DMSO control group, respectively. These results implied that DEAX and DEA inducing cytotoxicity was partially due to the MMP disruption in MDCK cells.

As shown in Figures 7(b)–7(d), the DCF fluorescence intensity significantly increased in a dose-dependent manner after adding DEAX or DEA to the medium. The DCF fluorescence intensity of the cells exposed to DEAX was approximately 9.15-12.1 times of that of the cells in the DMSO control group. Similarly, the amount of ROS produced in the DEA treatment group was 3.06–5.66 times of that of the DMSO control group. These data indicated that oxidative stress was an important pathway leading to MDCK cell apoptosis induced by DEAX or DEA.

## 4. Discussion

Out of concerns for the safety and efficacy of TCM, great efforts have been made to reveal the mechanisms behind the herbal drug-drug interaction [8, 23]. In this study, the cytotoxicity of both individual decoctions and three combined decoctions of RG and REP was systematically compared and the toxic components were traced, so as to elucidate the mechanism of RG-REP incompatibility and the possible toxic substances. Our results demonstrated that RG-REP incompatibility was conditionally established due to the RG dose-dependent REP toxicity change which was evidenced by MTT test, morphological observation, and caspase-3/7 assay. The toxicity of REP could be bidirectionally regulated by RG, and the combination ratio of both drugs was the determinant for incompatibility/compatibility. On the one hand, RG can unusually increase the toxicity of REP by inhibiting cell growth, deteriorating cell health status, and promoting cell apoptosis when it is used in nearly equal quantity with the latter and thereby justifies the incompatibility. On the other hand, the toxicity of REP can be attenuated by the further increased quantity of RG which supports the usual detoxification effect of RG and thus underlies the compatibility. Previous studies have not come to an agreement on the toxicity change resulted by concomitant use of RG and REP. Some researchers have proven that the toxicity of REP could be strengthened by RG [18, 24, 25], while others obtained the contrary conclusion based on their experiments [26–28]. Still, others have concluded that RG had no influence on the toxicity of REP [29, 30]. Although this issue remains unsettled, our research provided substantial evidence that RG plays a two-way regulatory effect on the toxicity of the RG-REP herb pair. The similar bidirectional regulation of RG was also observed when it was coused with another EIM associated herb, Flos Genkwa [31].

Someone may wonder why both incompatibility and compatibility simultaneously occurred when RG and REP were used together, i.e., 1 : 1 combination increased the toxicity while 2 : 1 and 3 : 1 combinations decreased toxicity. As usual, changes of components and toxicity induced by drug-drug interaction should be considered. To address this issue, we first compared the toxicity of the mixtures of individually decocted RG and REP at the ratios of 1 : 1, 2 : 1, and 3 : 1, respectively. The results (data were not shown) showed that the inhibition rates of the above three mixtures on MDCK cells had no significant difference compared with that of the REP decoction. This indicated that the key compound interaction from RG-REP may occur during the decoction process. Many researchers have found that when RG was decocted together with other herbs, natural compounds interacted with each other and led to their increased/decreased solubility and even generation of new substances accordingly [32–34]. This in turn increased/decreased the content and bioavailability of one or several substances and thus resulted in a synergistic/antagonistic effect [35, 36]. According to this theory, we compared the HPLC fingerprints of individual decoctions and combined decoctions to evaluate the composition change during the decoction preparation for the purpose of tracing toxic substances responsible for RG-REP incompatibility. As a result, the contents of DEA and DEAX in each decoction changed consistently with their toxic effect on MDCK cells. Therefore, RG should have an impact on the dissolution of both compounds, which may contribute to the toxicity change during RG-REP concomitant use.

To confirm that DEAX and DEA are definitely responsible for the toxicity change ascribed to RG-REP concomitant use, we performed validation experiments with these two compounds. The results showed that the compounds significantly decreased cell viability in a concentration-dependent manner and induced the cellular morphological changes described above. All of these were in agreement with the toxicity change of RG-REP decoctions, and thus, the roles of both compounds were confirmed. Literature [37] also reported that DEA could inhibit the proliferation of HepG2 cells, which provided a strong support for our conclusion.

The next question is how the toxicity was induced by the 1 : 1 RG-REP decoction, DEAX, and DEA. To this end, the percentages of living, apoptotic, and necrotic cells after treatment with the decoctions or compounds were analyzed. The results evidenced that apoptosis was the primary cell death manner induced by these substances. It has been reported that ROS is an important response to cellular injury and apoptotic cell death [22]. Excessive ROS impaired the integrity of the mitochondrial membrane, which subsequently caused MMP disruption, and eventually led to apoptosis through the mitochondrial apoptotic pathway [38]. Our results showed that the 1 : 1 RG-REP decoction, DEAX, and DEA resulted in remarkably decreased MMP (Figure 6), which was associated with the dysfunctional mitochondria. This tendency was positively correlated with cytotoxicity and ROS generation (Figure 7). What is more, ROS alternatively influences several cellular processes including cell cycle, cell differentiation, and apoptosis. However, this effect is a specific procedure depending on the amount and duration of ROS exposure [39]. In this study, we observed the invisible increment of the G0/G1 phase in the 1 : 1 RG-REP decoction group compared with the REP decoction group (Figure 8). It demonstrated that the cell cycle arrest might not play a key role in the apoptosis induced by RG-REP concomitant use. Thus, the ROS accumulation thereby induced was only sufficient for cell apoptosis increment, but insufficient for G0/G1 cell cycle arrest. Furthermore, ROS-mediated mitochondrial dysfunction could be associated with the increased cell apoptosis and eventually resulted in toxicity to MDCK cells. Previous studies also indicated that mitochondrial dysfunction caused by ROS overproduction is the main reason for nephrotoxicity induced by some drugs [40, 41].

## 5. Conclusions

The incompatibility of RG-REP concomitant use is conditionally established because of the bidirectional regulatory effect of RG. The major compounds responsible for RG-REP incompatibility are DEAX and DEA, which results in toxicity through activation of mitochondria-dependent apoptosis induced by the increased ROS production.

## Figures and Tables

**Table 1:** The ratios and doses of RG and REP in decoctions.

Decoctions	RG (g/L)	REP (g/L)	Ratio (RG : REP)
RG	4.8	—	—
REP	—	1.6	—
1 : 1 RG-REP	1.6	1.6	1 : 1
2 : 1 RG-REP	3.2	1.6	2 : 1
3 : 1 RG-REP	4.8	1.6	3 : 1

## Data Availability

The experimental data used to support the findings of this study are available from the corresponding author upon request.

## Supplementary Materials

Supplementary Materials Supplementary Figure 1: the influence of the RG-REP decoctions on the inhibition rate of MDCK cells. (a–c) show the inhibition rates of RG, REP, 0.5 : 1 RG-REP, 0.75 : 1 RG-REP, 1 : 1 RG-REP, 2 : 1 RG-REP, and 3 : 1 RG-REP on MDCK cells at a series of concentrations at 12, 24, and 48 h, respectively. All data are presented as means ± SD, *n* = 3. ^∗^0.01 < *P* < 0.05 and ^∗∗^*P* < 0.01, compared with the REP group.
