# Supplementary figures and images for: In Vitro Nephrotoxicity Induced by Herb-Herb Interaction between Radix Glycyrrhizae and Radix Euphorbiae Pekinensis
Source: Oxid Med Cell Longev. 2020 Apr 27;2020:6894751. doi: 10.1155/2020/6894751 (PMC7204103; doi:10.1155/2020/6894751)

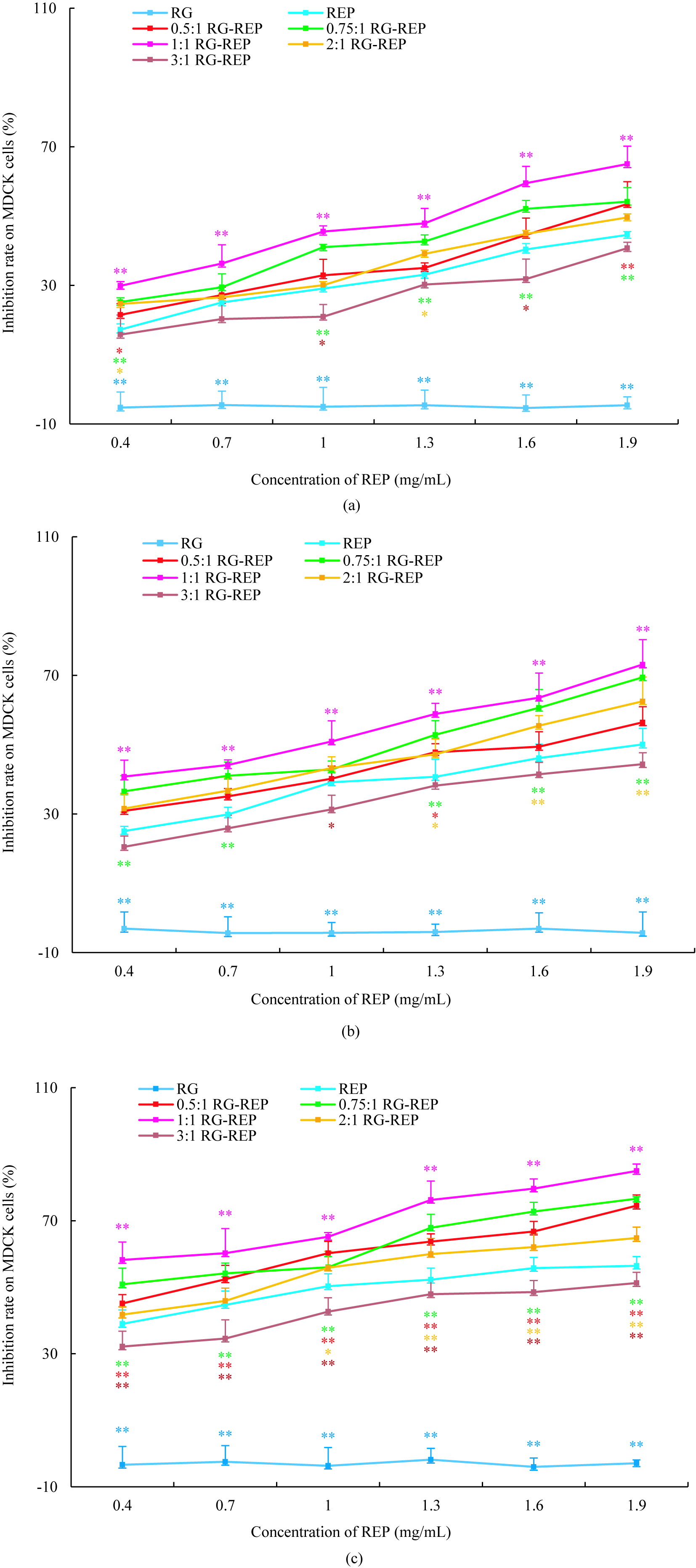

Supplement: Supplementary Materials — Supplementary Figure 1: the influence of the RG-REP decoctions on the inhibition rate of MDCK cells. (a–c) show the inhibition rates of RG, REP, 0.5 : 1 RG-REP, 0.75 : 1 RG-REP, 1 : 1 RG-REP, 2 : 1 RG-REP, and 3 : 1 RG-REP on MDCK cells at a series of concentrations at 12, 24, and 48 h, respectively. All data are presented as means ± SD, n = 3. ∗0.01 < P < 0.05 and ∗∗P < 0.01, compared with the REP group. [file 6894751.f1.zip › 6894751.f1/Supplementary Figure.tif]
